# Productivity and carbon fluxes depend on species and symbiont density in soft coral symbioses

**DOI:** 10.1038/s41598-019-54209-8

**Published:** 2019-11-28

**Authors:** Chloé A. Pupier, Maoz Fine, Vanessa N. Bednarz, Cécile Rottier, Renaud Grover, Christine Ferrier-Pagès

**Affiliations:** 1 Centre Scientifique de Monaco, Marine Department, 8 Quai Antoine Ier, MC-98000 Monaco, Principality of Monaco; 2 Sorbonne Université, Collège doctoral, F-75005 Paris, France; 3 The Mina and Everard Goodman Faculty of Life Sciences, Bar-Ilan University, Ramat-Gan, 52900 Israel; 4 The Interuniversity Institute for Marine Science in Eilat, PO Box 469, Eilat, 88103 Israel

**Keywords:** Stable isotope analysis, Animal physiology, Ecophysiology, Marine biology

## Abstract

Soft corals often constitute one of the major benthic groups of coral reefs. Although they have been documented to outcompete reef-building corals following environmental disturbances, their physiological performance and thus their functional importance in reefs are still poorly understood. In particular, the acclimatization to depth of soft corals harboring dinoflagellate symbionts and the metabolic interactions between these two partners have received little attention. We performed stable isotope tracer experiments on two soft coral species (*Litophyton* sp. and *Rhytisma fulvum fulvum*) from shallow and upper mesophotic Red Sea coral reefs to quantify the acquisition and allocation of autotrophic carbon within the symbiotic association. Carbon acquisition and respiration measurements distinguish *Litophyton* sp. as mainly autotrophic and *Rhytisma fulvum fulvum* as rather heterotrophic species. In both species, carbon acquisition was constant at the two investigated depths. This is a major difference from scleractinian corals, whose carbon acquisition decreases with depth. In addition, carbon acquisition and photosynthate translocation to the host decreased with an increase in symbiont density, suggesting that nutrient provision to octocoral symbionts can quickly become a limiting factor of their productivity. These findings improve our understanding of the biology of soft corals at the organism-scale and further highlight the need to investigate how their nutrition will be affected under changing environmental conditions.

## Introduction

Mutualistic symbioses between cnidarians and photosynthetic dinoflagellates of the family Symbiodiniaceae are widespread in marine environments^1^. In coral reef ecosystems, this association is the backbone for the growth and survival of corals surrounded by oligotrophic waters that hardly provide any exogenous nutrients. Indeed, the dinoflagellate symbionts of scleractinian corals translocate a large proportion of their photosynthetically fixed carbon compounds to the coral host for its own nutrition, growth, reproduction and energetic needs^2^. The percentage of carbon translocation can be as high as 90% in well-lit shallow waters^3,4^, and can remain high in mesophotic environments^5^. However, the total amount of autotrophically-acquired carbon generally decreases from shallow to deep reef environments through a reduced productivity of dinoflagellate symbionts^5^. Simultaneously, the coral host becomes more dependent on heterotrophic food sources and it is the plasticity between host heterotrophy and symbiont autotrophy that allows the association to thrive in such contrasting reef environments^6^. So far, our understanding of coral holobiont performance and host-symbiont metabolic interactions under different environmental conditions is limited to studies on scleractinian corals, while there is very little information available for other prominent members of reef systems such as soft corals (Alcyonacea).

Soft corals often constitute the second major benthic group of reef ecosystems^7^. They can thrive with relative high abundance and diversity under very different environmental conditions ranging from turbid to clear-water^8,9^ or from shallow to mesophotic reef environments^10^. Similarly to scleractinian corals, a large proportion (≥50%) of soft coral taxa is associated with Symbiodiniaceae in the Eastern Pacific, Caribbean, Red Sea and Great Barrier Reef^7^. There is evidence showing that the abundance of soft coral populations maintained or increased in most regions worldwide whereas scleractinian coral cover generally declined over the last decades^11,12^, due to increased sea surface temperatures, eutrophication and pollution. This is usually explained by a greater nutritional plasticity of soft corals, which are considered as mixotrophic species^13^. They can therefore acquire nutrients through the autotrophic activity of the symbionts, but also through the heterotrophic capacity of the host^8,13^. Evidence in the literature demonstrates that feeding has a positive effect on coral tissue, enhancing the growth of both partners of the symbiosis^14^. In addition, feeding can play a central role in maintaining physiological function when autotrophy is reduced^15^. The lower dependency of soft corals on autotrophy, compared to scleractinian corals, has been deduced from few measurements performed in shallow water conditions (0–20 m)^7,13,16–18^. Therefore, knowledge gaps exist for the main autotrophic physiological processes in soft corals thriving under different environmental conditions. The few studies which have dealt with their carbon budget observed highly variable contributions of photosynthetically fixed carbon provided by the symbionts to the host, with carbon translocation rates ranging from 10% in *Capnella gabonensis* to 75% in *Sinularia flexibilis*^19–22^. In addition, different normalization metrics for physiological processes in soft and scleractinian corals often hinder comparisons of the trophic characteristics between the two groups.

While tropical soft corals are considered reef engineers which create habitats for other reef species^7,23^, little comprehensive data exist on their trophic ecology, and therefore on their functional role as primary producers and carbon sinks within the reef ecosystem^8,13,24,25^. Since autotrophic carbon acquisition is a key factor shaping coral productivity, physiology, and ecology, and partly explains coral success or failure under changing environmental conditions, more studies should be dedicated to better understand the autotrophic capacity of soft corals. Therefore, the present study aims to assess the carbon fluxes between dinoflagellate symbionts and host of two common soft coral species (*Litophyton* sp. and *Rhytisma fulvum fulvum*) from shallow and mesophotic reefs of the Gulf of Eilat. For this purpose, we used stable isotope tracers (^13^C-bicarbonate) to measure the rates of carbon fixed, exchanged and lost by the shallow and mesophotic symbiotic associations under their natural photosynthetically active irradiance (PAR) levels. Importantly, we also compare our results with those of scleractinian coral species by applying a standardized normalization metric for all parameters^26,27^.

## Results

### Physiological measurements

**Figure 1:**
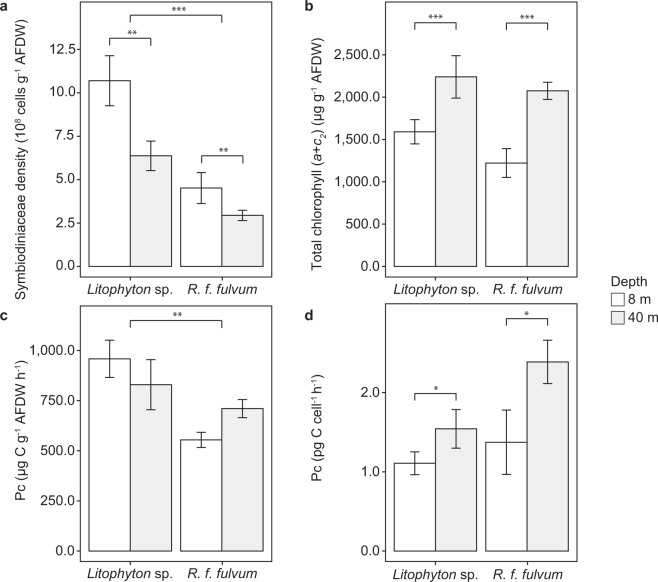
Physiological and tissue descriptors measured in the soft coral species investigated. For panels (**a–b**) and (**c**), data are normalized to ash-free dry weight (AFDW). (**a**) Symbiodiniaceae density. (**b**) Total chlorophyll concentration. (**c**) Autotrophic carbon acquisition. (**d**) Autotrophic carbon acquisition per Symbiodiniaceae cell. Error bars represent standard error. There were significant differences between species and depths for (**a**), between depths for (**b–d**), and between species for (**c**) (*p < 0.05, **p < 0.01, ***p < 0.001).

The dinoflagellate-host association was species-specific. *Litophyton* sp. harbored *Symbiodinium* sp. (formerly *Symbiodinium* clade A^28^) whereas *R. f. fulvum* was associated with *Cladocopium thermophilum* (formerly *Symbiodinium thermophilum* clade C3^28,29^), regardless of the depth investigated. Symbiodiniaceae density, normalized to AFDW, was two-fold higher in *Litophyton* sp. than in *R. f. fulvum* at both depths and was for both species always higher in shallow than in mesophotic con-specifics (Fig. 1a, Table S1). For both species, total chlorophyll concentrations (per AFDW) were significantly higher for mesophotic corals (Fig. 1b, Table S1). Also, symbionts contained significantly more chlorophyll per cell in mesophotic corals (2.8·10^−6^ ± 3.2·10^−7^ µg cell^−1^ for *Litophyton* sp. and 7.5·10^−6^ ± 4.9·10^−7^ µg cell^−1^ for *R. f. fulvum*, Table S1) as compared to shallow corals (1.6·10^−6^ ± 1.5·10^−7^ µg cell^−1^ for *Litophyton* sp. and 4.2·10^−6^ ± 1.2·10^−6^ µg cell^−1^ for *R. f. fulvum*, Table S1).

Autotrophic carbon acquisition, estimated using ^13^C-bicarbonate incorporation over 5 h under maximal irradiance (P_C_, expressed in µg C g^−1^ AFDW h^−1^), was significantly different between the two coral species, with *Litophyton* sp. exhibiting higher Pc rates than *R. f. fulvum* (Fig. 1c, Table S1). When normalized to symbiont cells, Pc rates were significantly different between depths, with higher values obtained in mesophotic conditions (Fig. 1d, Table S1). Respiration rates (Rc, expressed in µg C g^−1^ AFDW h^−1^) were similar between depths for *R. f. fulvum* (347 ± 45 and 489 ± 47 in shallow and mesophotic conditions, respectively), whereas they were two-fold lower for mesophotic (148 ± 47) than shallow (406 ± 18) nubbins of *Litophyton* sp. (Table S1, Tukey HSD p < 0.01). While symbiont respiration accounted for less than 1% of the holobiont respiration for *Litophyton* sp. in both depths, symbiont respiration accounted ca. 6% and 4% to holobiont respiration for mesophotic and shallow *R. f. fulvum*, respectively.

### Carbon budget

**Figure 2:**
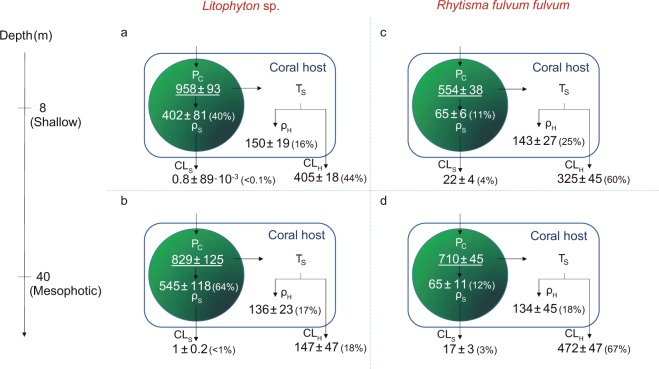
Carbon budgets obtained after the 5 hours pulse period in the soft coral species investigated. (**a**) Shallow *Litophyton* sp. (**b**) Mesophotic *Litophyton* sp. (**c**) Shallow *Rhytisma fulvum fulvum*. (**d**) Mesophotic *Rhytisma fulvum fulvum*. Symbionts are represented by a green circle. P_C_ = autotrophic carbon acquisition. ρ_S_ = carbon assimilated in Symbiodiniaceae. ρ_H_ = carbon assimilated by the coral host. T_S _ = translocation of photosynthates. CL_S_ = carbon lost by the symbionts through respiration. CL_H_ = carbon lost by the host through respiration. P_C_ and carbon fluxes are expressed in µg C g^−1^AFDW h^−1^.

After the pulse period (5 h), both species exhibited significant differences in their carbon budget between depths (Fig. 2a–d, Tables S1 and S2). Overall, rates of photosynthate translocation were lower in *Litophyton* sp. at both depths (36% to 60% of the total fixed carbon) compared to *R. f. fulvum* (85%), and symbionts of *Litophyton* sp. retained four to five times more carbon than those of *R. f. fulvum* (Fig. 2b–d, Table S1). However, no difference was observed in the rate of carbon assimilated by the host between depths and species (134–150 µg C g^−1^ AFDW h^−1^). For *Litophyton* sp. from mesophotic depth, symbionts translocated less carbon to the host (36% versus 60%) and less carbon was lost by the holobiont (19% versus 44%) as compared to nubbins from shallow depth. On the contrary, photosynthate translocation (85%) and carbon loss (64–70%) were comparable between shallow and mesophotic *R. f. fulvum* corals.

**Figure 3:**
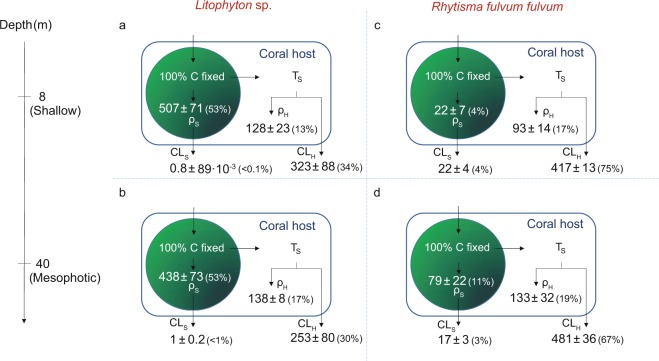
Carbon budgets obtained after the 24 hours chase period in the soft coral species investigated. (**a**) Shallow *Litophyton* sp. (**b**) Mesophotic *Litophyton* sp. (**c**) Shallow *Rhytisma fulvum fulvum*. (**d**) Mesophotic *Rhytisma fulvum fulvum*. Symbionts are represented by a green circle. ρ_S_ = carbon assimilated in Symbiodiniaceae. ρ_H_ = carbon assimilated by the coral host. T_S_ = translocation of photosynthetates. CL_S_ = carbon lost by the symbionts through respiration. CL_H_ = carbon lost by the host through respiration and mucus production. Carbon fluxes are expressed in µg C g^−1^AFDW h^−1^.

After the chase period (24 h), the carbon budget of both species was clearly species-specific but without depth-specific differences (Fig. 3a–d, Table S1). In *Litophyton* sp., symbiont cells assimilated half of the photosynthesized carbon and translocated only 47% to the coral host in both shallow and mesophotic colonies (Fig. 3a–d, Table S1). On the contrary, symbionts in *R. f. fulvum* translocated 86–92% of the photosynthetically fixed carbon to the host and assimilated only 4–11% of this carbon for their own energetic needs. Consequently, significant differences were evident between the two species for the rates of carbon 1) translocated (up to one third lower in *Litophyton* sp. than in *R. f. fulvum*), 2) retained in the symbionts (ten-fold higher in *Litophyton* sp. than in *R. f. fulvum*) and 3) lost from the symbiotic association (two-fold lower in *Litophyton* sp. than in *R. f. fulvum*) (Fig. 3a–d, Table S1).

### Relationship between Symbiodiniaceae density and carbon flux

**Figure 4:**
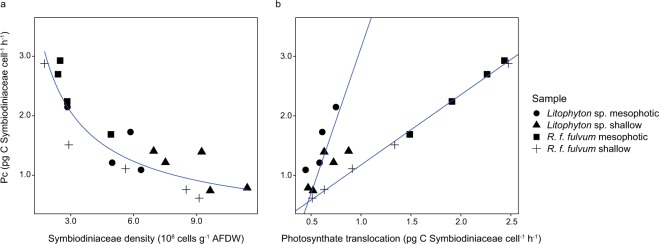
Relationships between Symbiodiniaceae density and carbon fluxes of the soft coral species investigated. (**a**) Exponential relationship between carbon acquisition (P_C_) per cell and density of Symbiodiniaceae cells of *Litophyton* sp. and *Rhytisma fulvum fulvum* (R² = 0.78, y = 4.7087x^−0.744^). (**b**) Orthogonal regressions between carbon acquisition (P_C_) per cell and photosynthates translocation of Symbiodiniaceae cells of *Litophyton* sp. (R² = 0.60, y = 4.8964x − 1.7547) and *Rhytisma fulvum fulvum* (R² = 0.99, y = 1.1818x − 0.0091). AFDW = ash-free dry weight.

Our results show that in the two coral species at both depths the carbon acquisition per symbiont cell exponentially decreased with increasing symbiont density (Fig. 4a). In addition, we found a positive linear correlation between symbiont cell carbon acquisition and translocation (Fig. 4b).

## Discussion

This study shows that the functioning of the coral-dinoflagellate symbiosis in soft corals holds some different characteristics to that in many scleractinian corals, and that the two groups are differently acclimatized to depth. The two investigated soft coral species maintained an equivalent carbon acquisition from shallow to poorly-lit mesophotic habitats, while carbon acquisition of scleractinian corals generally decreases with depth. This autotrophic efficiency, combined with putative high heterotrophic capacities, may provide soft corals with an ecological advantage in the upper Red Sea mesophotic reefs^10^. In addition, the results show that carbon acquisition and translocation rates per symbiont cell were negatively correlated with the symbiont density within the host tissue, with a higher assimilation of carbon in symbiont biomass under high symbiont density. However, the rates at which carbon was translocated to and assimilated by the host were unaffected, indicating that dense symbiont populations can remain mutualistic.

**Figure 5:**
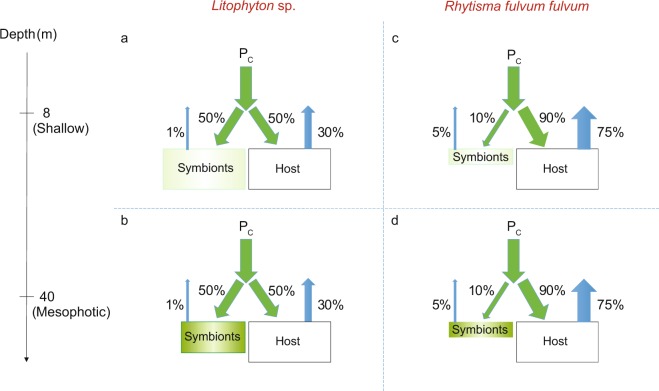
Metabolic interactions between soft corals and their dinoflagellate symbionts. (**a**) Shallow *Litophyton* sp. (**b**) Mesophotic *Litophyton* sp. (**c**) Shallow *Rhytisma fulvum fulvum*. (**d**) Mesophotic *Rhytisma fulvum fulvum*. Boxes and arrows are indicative of differences in density and fluxes. Color intensity of symbionts corresponds to chlorophyll concentration levels. P_C_ = autotrophic carbon acquisition.

Our findings indicate that the mean autotrophic carbon acquisition in both soft coral species (measured during 5 h and using ^13^C-labelling) remained stable from shallow to mesophotic environments, which suggests that soft corals are either photo-limited in shallow waters or well photo-acclimatized to depth (Fig. 5). This is in agreement with previous observations^13^ that maximal photosynthetic rates of several soft coral species from the Great Barrier Reef were measured at 20 m depth rather than in shallower waters (Table 1). Such constant carbon acquisition along the depth gradient is surprising in comparison to the general patterns observed in scleractinian corals. Most scleractinian corals experience a significant decrease in rates of carbon acquisition with depth, although this physiological parameter has been poorly investigated in mesophotic environments^5,30,31^. However, a recent study, which investigated the photosynthetic performance of the mesophotic coral *Euphyllia paradivisa*, found a high photosynthetic capacity of this coral species under low light conditions^32^.

**Table 1:** Tissues descriptors and autotrophic carbon acquisition reported for different octocorals, normalized to ash-free dry weight (AFDW).

OCTOCORALS					
Species	Depth	Symbiont density	Total chlorophyll (a + c_2_)	P_C_	Reference
	(m)	(10^8^ cell g^−1^ AFDW)	(µg g^−1^ AFDW)	(µg C g^−1^ AFDW h^−1^)	
**Great Barrier Reef**					
*Asterospicularia* sp.	20			530	Fabricius and Klumpp (1995)
*Briareum stechei*	5			419–452	
	20			707–838	
*Capnella lacertiliensis*	5			949–1047	
	20			511	
*Efflatounaria* sp.	5			975–1375	
	20			772–1191	
*Lobophytum spp*.	5			367	
	20			419	
*Nephthea sp*.	5			1145	
	10			1100	
	20			956	
*Paralemnalia sp*.	5			838–923	
	10			792–1191	
	20			884–956	
*Sarcophyton spp*.	5			367–445	
	10			1034–3076	
	20			511–707	
*Sinularia spp*.	5			713–825	
	10			772	
	20			406–903	
*Xenia spp*.	5			890–1231	
	10			1165–1872	
	20			812	
**Caribbean**					
*Antillogorgia sp*.	2–8	1–3	1300–2120	1105–2291	Baker *et al*. (2015); Rossi *et al*. (2018)
*Briareum asbestinum*	3–8			249	Baker *et al*. (2015)
*Eunicea sp*.	2–8	1–4	760–2150	51–382	Baker *et al*. (2015); Rossi *et al*. (2018)
*Gorgonia ventalina*	2–8	1	730	457–3284	
*Plexaura sp*.	2–8	0.5	1280	20–742	
*Pterogorgia anceps*	2–8	2	3490–4050	184–3338	
**Red Sea**					
***Litophyton*** **sp**.	**8**	**9.4**	**1591**	**958**	**This study**
	**40**	**6.4**	**2240**	**829**	
***Rhytisma f. fulvum***	**8**	**4.5**	**1222**	**554**	
	**40**	**2.9**	**2075**	**710**	

P_C_ = autotrophic carbon acquisition.

Pigment content, symbiont density and symbiont genus are generally involved in shaping the photo-acclimatization of the coral symbiosis from shallow to mesophotic environments. Our results highlight for the two soft coral species a host-specific Symbiodiniaceae genus that remained stable along the depth profile up to 40 m depth. *Litophyton* sp. was associated with *Symbiodinium* sp., as previously reported (formerly *Symbiodinium* clade A^33,34^), while *R. f. fulvum* was rather associated with *Cladocopium* sp., in agreement with studies performed in the Red Sea (formerly *Symbiodinium* clade C^33^), or the Great Barrier Reef^35,36^. Such clade specificity is assumed to be persistent both in space and time in octocorals^37,38^. Thus, acclimatization of the soft corals to low light levels was independent of the symbiont genus, but potentially driven by higher concentrations of total chlorophyll per AFDW and per symbiont cell in mesophotic corals (Fig. 1b), which maximize the light harvesting capacity of the cells under low light levels^5,39^. In addition, both species exhibited a decreased symbiont density in mesophotic as compared to shallow colonies which is likely another adaptation to depth by reducing self-shading under reduced PAR^40,41^. A lower symbiont density also reduces the competition for inorganic nutrient supply^42^. It has been demonstrated that mesophotic scleractinian corals can display several strategies to acclimatize to low light levels, such as modifications in the organization of photosynthetic apparatus (shift to a PSII-based system, including additional photosynthetic antenna^30^), modifications in the organization of antenna (growth of specialized paracrystalline light-harvesting antenna domains^43^), or the synthesis of fluorescent pigments by the host (that can perform wavelength transformation to facilitate light penetration^44^); however, these strategies remain to be further investigated in soft corals.

The autotrophic carbon acquisition to respiration ratios (P_C_/R_C_) of *Litophyton* sp. ranged from 1 to 2 from shallow to mesophotic depths, the latter value being above the conservative threshold for net autotrophy (P_C_/R_C_ > 1.5). Considering that the solar radiation level in November is one of the lowest annual levels^45^, the data suggest that *Litophyton* sp. is mostly an autotrophic species. On the contrary, P_C_/R_C_ ratios of *R. f. fulvum* in November were always below compensation (P_C_/R_C_ = 1) even when considering a maximal autotrophic carbon acquisition rate sustained for 12 h. This strongly suggests that this species relies, at least in winter, on heterotrophy to sustain its daily respiratory needs. The difference in P_C_/R_C_ between the two species may be due to their different growth shapes and different micro-morphological features of the polyps. Two recent studies on gorgonian octocorals, indeed highlighted a correlation between host morphology, polyp size and productivity^17,18^, thereby corroborating the first observations of Porter (1976)^46^.

Symbiont cell carbon acquisition was negatively correlated with symbiont density in both investigated soft coral species and decreased exponentially as soon as the symbiont density increased. Although this pattern is first described on soft corals in this study, similar observations were reported with gorgonian octocorals^18^ and scleractinian corals^47^, and might be due to a host-dependent regulation of light and nutrient ressources^47^, or a self-shading of the symbionts^42^.

Our results also highlight a positive correlation between carbon acquisition and carbon translocation rates per symbiont cell, similarly to a previous study on scleractinian corals^48^. In *Litophyton* sp., a low carbon acquisition per cell corresponded to a high symbiont density and consequently, symbiont cells assimilated more carbon for their own metabolism rather than translocating it to the host (50% translocation). In contrast, the symbionts in *R. f. fulvum* translocated significantly more carbon (80 to 90% translocation) to the host, likely due to their lower abundancy and increased rates of carbon acquisition per cell. Carbon translocation rates of >80% have also been reported for other coral species^2–4^. For example, Scheufen *et al*.^47^ measured highest productivity rates in corals during the very oligotrophic summer season despite seasonally reduced symbiont density. This may also imply a high carbon translocation to the host that is essential when exogenous food sources are scarce.

Differences in cell-specific carbon assimilation and translocation rates may not only be related to the symbiont density but also to the symbiont genus. Indeed, *Symbiodinium* sp. (associated with *Litophyton* sp. here) is known to translocate less carbon to its host as compared to *Cladocopium* sp. (associated with *R. fulvum fulvum* here)^48–50^. However, an important observation is that the lower percentage of carbon translocation in *Litophyton* sp. is not indicative of a shift towards symbiont parasitism^50^, as the carbon translocation to the host still exceeded the metabolic costs for holobiont respiration. In *R. f. fulvum*, due to the low symbiont density, an increased translocation rate per cell was needed to transfer the same amount of carbon to the host. In addition, the percentage of carbon lost by the host was lower in *Litophyton* sp. than in *R. f. fulvum*, compensating for the lower percentage of carbon translocated from the symbionts. As a consequence, the rates at which carbon was retained in the host were constant between species and depths (between 13% and 19% of the photosynthesized carbon, or 93 to 138 µg C g^−1^ AFDW h^−1^). Assimilation rates of 10 to 20% of the photosynthesized carbon by the animal host seems to be a general feature in corals^4,5^.

Soft and scleractinian corals therefore seem to exhibit a different trend of carbon acquisition along the depth gradient, although more investigations with different scleractinian and soft coral species are still needed. In order to compare carbon fixation per biomass between soft and scleractinian corals, we used data previously obtained on the scleractinian coral *Stylophora pistillata* sampled at the exact same location, depths and month^5^ (Table S3) and normalized the rates to AFDW. The estimates we obtained show that soft corals fix less carbon per biomass as compared to *S. pistillata* at shallow depth in November (estimation of 2426 µg C g^−1^ AFDW h^−1^ for *S. pistillata* versus 554–958 µg C g^−1^ AFDW h^−1^), although soft corals contain comparable or higher dinoflagellate density^51^ and chlorophyll content per biomass (Table S3) than scleractinian corals. However, in deeper environments, soft corals tend to fix carbon per biomass at similar or even higher rates (estimation of 555 µg C g^−1^ AFDW h^−1^ for *S. pistillata* versus 710–829 µg C g^−1^ AFDW h^−1^ for *R. f. fulvum* and *Litophyton* sp.). This may indicate that in shallow waters, soft corals have particularly less fixed carbon available to maintain or grow their biomass as compared to scleractinian corals. Morphological characteristics of the host, which have an impact on the diffusion and transmission of light to the symbionts, may explain differences in carbon acquisition in shallow water corals. Soft corals lack a calcium carbonate skeleton, contrary to scleractinian corals, and only possess sclerites as calcified structures. However, skeletons present light scattering abilities, which enhance light absorption efficiency of the symbionts (e.g., Enríquez *et al*.)^52^. Although characteristics of sclerites and tissues remain to be investigated to understand the potential light amplification and dispersion in soft corals, we hypothesized that they would affect the light environment of the symbionts to a lesser extent than skeletons do in scleractinian corals. In addition, soft corals have a thick coenenchyme and exhibit a low ratio of colony surface area to volume, which does not favor light exposure and gas or nutrient exchange through the epidermal tissue^53^. Finally, the variability of their hydroskeleton allows soft corals to contract and expand their tissue. Tissue contraction removes symbionts from the tissue surface, thereby higher irradiance is required to reach the symbionts and to achieve photosynthetic compensation and saturation.

Understanding nutritional ecology of octocorals is still in its infancy. The common belief is that octocorals are more heterotrophic than scleractinian corals, because of their low carbon acquisition in surface waters, and because their morphology is more suited for heterotrophy^7^. Two recent studies on Caribbean gorgonian octocorals have observed a possible correlation between host morphology and symbiont performance^17,18^. Octocorals with thin branches and small polyps can be more autotrophic as compared to those with a massive shape and large polyps. In our work, a different degree of autotrophy was demonstrated for the two investigated soft coral species, which might be linked to morphological traits. The arborescent shape of *Litophyton* sp. may enhance the exposure of symbionts to light and thus favour autotrophy compared to the mat-forming shape of *R. f. fulvum*. In addition, our results strongly support the view that high symbiont density results in lower cell-specific carbon translocation and acquisition. However, soft corals with high symbiont densities (*Litophyton* sp.) reached similar carbon translocation than those with low symbiont density. Further work involving various soft coral species, growth-shapes and symbiont-specificities will be essential to provide more insight into the physiology and trophic ecology of octocorals thriving along environmental gradients.

## Material and Methods

### Soft coral collection and experimental setup

The study was conducted in November 2017 at the Inter-University Institute for Marine Science (IUI), Gulf of Eilat. Experiments were performed with two soft coral species harboring dinoflagellate symbionts, *Litophyton* sp. (Forskål, 1775) and *Rhytisma fulvum fulvum* (Forskål, 1775) belonging to the Alcyoniidae family.

Ten colonies per species were sampled on the reef adjacent to the IUI (29°51′N, 34°94′E) at 8 m and 40 m depth, respectively, to generate a total of 40 nubbins per species (two nubbins per colony). Nubbins were allowed to recover for ten days in open-water cages placed at the original collection site before being brought back to the Red Sea Simulator facility^54^. There, nubbins were allocated to three different outside aquaria per species and depth. All aquaria were continuously supplied with water directly pumped from the reef and were exposed to the natural diurnal light cycle. Furthermore, aquaria were shaded with several layers of mesh clothes to adjust daily maximum light levels to the maximum irradiance measured at the corresponding depth. Thus, all nubbins were maintained under natural diurnal variation in irradiance and received the depth-corresponding *in situ* maximum irradiance at noon. Levels of photosynthetically active radiation (PAR) were obtained from the Israel National Monitoring program of the Gulf of Eilat (http://www.iui-eilat.ac.il/Research/NMPMeteoData.aspx). During the five days of our experiment, surface water PAR reached 1200 µmoles photon m^−2^ s^−1^ at midday (Fig. S1). The PAR received at 10 m and 40 m depth peaked at ca. 550 µmoles photon m^−2^ s^−1^ and 50 µmoles photon m^−2^ s^−1^ respectively, considering attenuation coefficients of 0.072–0.1 m^−1^ at this precise location^55,56^. These values are in agreement with one-off measurements performed with a data logger during coral collection. The *in situ* seawater temperature (24 °C) and nutrient concentrations (<0.5 µM dissolved nitrogen, and  <0.2 µM dissolved phosphorus) were stable throughout the investigated depth gradient and period of time (http://www.iui-eilat.ac.il/Research/NMPMeteoData.aspx). Coral nubbins were maintained for one day under these conditions before the following measurements were performed in the outside aquaria under exactly the same environmental conditions.

### NaH^13^CO_3_ incubations

The rates at which carbon was fixed by the shallow and mesophotic coral colonies under their natural PAR levels were estimated using ^13^C- labelled bicarbonate according to Tremblay *et al*.^4^. To take into account variation in PAR levels during the day, corals were incubated for 5 h, between 10 am and 3 pm, to cover the maximal daily irradiance^45^ (Fig. S1). For each species and depth condition, 10 coral nubbins, from 10 different colonies were placed in individual beakers filled with 200 mL FSW enriched with 0.6 mM NaH^13^CO_3_ (98 atom % ^13^C, #372382, Sigma-Aldrich, St-Louis, MO, USA). After this “pulse” period of 5 h, half of the corals were sampled and stored frozen at −20 °C until further analysis (T_0_). The other half was transferred into 200 mL of non-enriched FSW for a chase period of 19 h (T_24_). The determination of %^13^C enrichment, and total carbon content in the symbionts and host compartments were performed with a Delta plus Mass spectrometer coupled to a C/N analyser (Thermo Fischer Scientific, Bremen, Germany). The natural isotopic abundance of each species at each depth was determined from the corals used for the respiration measurements. Detailed calculations are described in Tremblay *et al*.^4^. Data were normalized to ash-free dry weight of the organisms^27^.

### Physiological and tissue descriptor measurements

Estimation of the dark respiration rates of the whole colony, as well as of the isolated symbionts, was needed for the establishment of the daily carbon budget. Dark respiration rates were measured on four nubbins per species and depth (from four different colonies), as well as on freshly isolated symbionts from four other nubbins according to Tremblay *et al*.^4^. Dark-acclimated nubbins/symbionts were individually placed in stirred incubation chambers filled with 0.45 µM filtered seawater and maintained at 24 °C. Changes in dissolved oxygen were monitored over 30 minutes using optodes connected to an Oxy-4 (PreSense, Regensburg, Germany). Oxygen fluxes were converted into carbon equivalents (R_C_)^4^. The autotrophic carbon acquisition to respiration ratios (P_C_/R_C_) were estimated considering a maximal autotrophic carbon acquisition rate for 6 h, and a half rate for the remaining 6 h. At the end of the incubations, nubbins were flash-frozen in liquid nitrogen, freeze-dried, weighed for the total DW determination and processed as described in Pupier *et al*.^27^ (Supplementary information) for the further determination of the symbiont density, total chlorophyll concentration and ash-free dry weight (AFDW). The genus of the symbionts hosted by the shallow and mesophotic populations of *Litophyton* sp. and *R. f. fulvum* was investigated following the protocol of Santos *et al*.^57^.

### Interspecies comparisons

To compare our data on soft corals, normalized to AFDW, with those found in the literature on scleractinian corals, often normalized to skeletal surface area, it was necessary to use the same normalization metric. As surface area could not be measured with accuracy on soft corals (since they have a highly variable hydroskeleton which expands and shrinks the colony size by several folds depending on the environmental conditions), we normalized all data to AFDW^27^. For this purpose, we chose eight nubbins (skeletal surface area ranging from 4 to 29 cm²) of the scleractinian coral *Stylophora pistillata* originating from the Red Sea and grown at the Monaco Scientific Center, as it is one of the dominant species in the Gulf of Eilat and many data have been acquired on this species. AFDW was determined as described above after separating the tissue from the skeleton and combusting it. Surface area was determined using the wax technique^58^. A conversion factor (255.65 ± 8.98, Table S4), estimated from the ratio between skeletal surface area (in cm²) and AFDW (in g) of these nubbins, was used to transform data normalized to surface area (Table S3) into data normalized to AFDW.

### Statistical analyses

Analyses were performed using R software (R Foundation for Statistical Computing). All data were expressed as mean ± standard error. Prior to analyses, outlier values were identified using Grubb’s test and were excluded when p-values were significant (p < 0.05). Assumptions of normality and homoscedasticity of variance were evaluated through Shapiro’s and Bartlett’s tests along with graphical analyses of residuals. A two-way analysis of variance (ANOVA) was performed to test the effect of species and depth on symbiont density, chlorophyll concentrations and carbon acquisition. The rates at which carbon was assimilated, translocated and lost were analysed separately between the two time points (i.e., pulse and chase periods) using 2-way ANOVAs with species and depth as fixed effects. When the interaction was significant pairwise, Tukey tests were performed as *a posteriori* testing.

## Supplementary information

Supplementary Information
