# Supplementary material for: Productivity and carbon fluxes depend on species and symbiont density in soft coral symbioses
Source: Sci Rep. 2019 Nov 28;9:17819. doi: 10.1038/s41598-019-54209-8 (PMC6882883; doi:10.1038/s41598-019-54209-8)
Supplement: Supplementary file 1 — Supplementary Information [file 41598_2019_54209_MOESM1_ESM.pdf]

Supplementary Information

Productivity and carbon fluxes depend on species and symbiont density in soft coral symbioses

Chloé A. Pupier, Maoz Fine, Vanessa N. Bednarz, Cécile Rottier, Renaud Grover, Christine Ferrier-Pagès

Material and Methods

Physiological and tissue descriptor measurements

For the determination of the symbiont density, total chlorophyll concentration and ash-free dry weight (AFDW) from the experimental nubbins, we followed the protocol of Pupier et al.[1]. Briefly, freeze-dried samples were homogenized in a Potter tissue grinder. A subsample was weighed, and then combusted at 450°C for 4 h in a muffle furnace (Thermolyne 62700, Thermo Fischer Scientific, the United States). AFDW was determined as the difference between the DW and ash weight (AW) of the subsample and extrapolated to the total weight of the nubbin. The remaining sample was also weighed, and then homogenized with 10 mL distilled water (DI). The homogenate was centrifuged for 10 min (at 11,000 g at 4°C) to separate the animal host (supernatant) from the symbionts (pellet). Light microscopy confirmed the total removal of symbionts from the supernatant. The symbiont pellet was rinsed twice to eliminate any remaining host cells[2], and re-suspended in 10 mL DI. After mixing, 500 µL and 2.5 mL were subsampled for the determination of symbiont density and total chlorophyll concentration, respectively. Symbiont density was quantified microscopically via eight replicate haemocytometer counts (Neubauer-improved haemocytometer, Marienfeld, Germany). For chlorophyll analysis, the 2.5 mL subsamples were centrifuged for 10 min (at 8,000 g at 4°C) and the supernatant was discarded. Pellets were re-suspended into 4 mL of acetone (100%) amended with magnesium chloride (Sigma Aldrich, Germany) in order to extract the chlorophyll during 24h in the dark at 4°C. Chlorophyll a and c_2 concentrations were determined following the method of Jeffrey & Humphrey[3] by the use of a spectrophotometer (SAFAS, Monaco). Data were normalized to the total AFDW of the nubbin.

$NaH^{13}CO_3$ incubations

Carbon incorporation rates in the symbionts (ρ_S) and coral host tissue (ρ_H) were calculated as follow:

$$\rho = \frac{(C_{meas} - C_{nat}) * M_{sample} * M_c}{(C_{inc} - C_{meas}) * (t_{pulse} + t_{chase}) * AFDW}$$

where C_{meas} and C_{nat} are the percentages of ^{13}C measured on the samples (symbionts or host tissues) from ^{13}C-enriched experiments and controls, respectively. C_{inc} is the percent ^{13}C enrichment of the incubation medium; M_{sample} is the mass of the freeze-dried sample (mg); M_c is the mass of carbon per milligram of symbiont or host tissue (µg mg^{-1}); AFDW is the ash-free dry weight (g); and t_{pulse} and t_{chase} are incubation times (h) of the nubbins in the enriched and non-enriched incubation media, respectively. C_{inc} varied during the pulse chase and was calculated as:

$$C_{inc} = \frac{(C_{pulse} * t_{pulse}) + (C_{chase} * t_{chase})}{(t_{pulse} + t_{chase})}$$

where C_{pulse} and C_{chase} are the percent ^{13}C enrichment of the enriched and non-enriched incubation media, respectively (C_{chase}=1.1%).

The amount of carbon acquired through photosynthesis, translocated from the symbionts to the host (T_S) was calculated as:

$$T_S = P_C - \rho_S - R_S$$

where P_C is the total amount of autotrophic carbon produced by the symbionts; ρ_s is the carbon assimilation rate and R_S the respiration rate of the symbionts.

Finally, the amount of carbon lost (C_L) through respiration (R_C) and through particulate and dissolved carbon release ($\rho_{POC/DOC}$) is:

$$C_L = P_C - \rho_S - \rho_H$$

Supplementary tables

Table S1: Statistical results (two-way ANOVA) about the effect of species and depth on tissue descriptors (Symbiodiniaceae density and total chlorophyll (a+c_2) concentrations, *n* = 9 to 10 per condition) and carbon budgets in *Litophyton* sp. and *Rhystima fulvum fulvum* from 8 m and 40 m depth (*n* = 3 to 5 per condition). AFDW = ash-free dry weight. P_C = autotrophic carbon acquisition. Rc = respired carbon. Pulse and chase refer to the periods of time to trace the fate of inorganic carbon assimilated by host (ρ_H) and symbionts (ρ_S). T_S = photosynthates translocation. C_L = carbon lost as respiration of the holobiont and organic matter release.

		Df	F-value	p-value
**TISSUE DESCRIPTORS**				
Symbiodiniaceae density				
	Species	1	31.230	**< 0.01**
	Depth	1	9.783	**< 0.01**
	Species:Depth	1	0.996	0.32518
	Residuals	35		
Chlorophyll concentration (µg g^{-1} AFDW)				
	Species	1	2.326	0.135965
	Depth	1	18.395	**< 0.01**
	Species:Depth	1	0.341	0.563096
	Residuals	36		
Chlorophyll concentration (µg symbiont $cell^{-1}$)				
	Species	1	27.996	**< 0.01**
	Depth	1	10.260	**< 0.01**
	Species:Depth	1	2.188	0.14806
	Residuals	35		
Pc (µg C g^{-1} AFDW h^{-1})				
	Species	1	9.619	**< 0.01**
	Depth	1	0.004	0.9524
	Species:Depth	1	2.654	0.1241
	Residuals	15		
Pc (µg C symbiont $cell^{-1}$)				
	Species	1	3.231	0.0938
	Depth	1	6.124	**0.0267**
	Species:Depth	1	0.978	0.3395
	Residuals	14		
Rc (µg C g^{-1} AFDW h^{-1})				
	Species	1	13.504	**< 0.01**
	Depth	1	3.151	0.10352
	Species:Depth	1	23.537	**< 0.01**
	Residuals	11		
**PULSE**				
ρ_H (µg C g^{-1} AFDW h^{-1})				
	Species	1	0.020	0.888
	Depth	1	0.177	0.680
	Depth:Species	1	0.008	0.931

	Residuals	15		
ρ_S (µg C g^{-1} AFDW h^{-1})				
	Species	1	27.360	< **0.01**
	Depth	1	1.285	0.274752
	Depth:Species	1	0.623	0.442087
	Residuals	15		
T_S (µg C g^{-1} AFDW h^{-1})				
	Species	1	15.25	< **0.01**
	Depth	1	7.95	**0.01294**
	Depth:Species	1	52.82	< **0.01**
	Residuals	15		
C_L (µg C g^{-1} AFDW h^{-1})				
	Species	1	78.426	< **0.01**
	Depth	1	6.758	**0.0201**
	Depth:Species	1	14.945	< **0.01**
	Residuals	15		
**CHASE**				
ρ_H (µg C g^{-1} AFDW h^{-1})				
	Species	1	0.908	0.357
	Depth	1	1.309	0.272
	Depth:Species	1	0.515	0.485
	Residuals	14		
ρ_S (µg C g^{-1} AFDW h^{-1})				
	Species	1	51.692	< **0.01**
	Depth	1	0.052	0.823
	Depth:Species	1	1.147	0.302
	Residuals	14		
T_S (µg C g^{-1} AFDW h^{-1})				
	Species	1	5.850	**0.0298**
	Depth	1	0.049	0.8277
	Depth:Species	1	1.935	0.1859
	Residuals	14		
C_L (µg C g^{-1} AFDW h^{-1})				
	Species	1	6.785	**0.0208**
	Depth	1	0.033	0.8584
	Depth:Species	1	0.866	0.3678
	Residuals	14		

Table S2 Atom% ^{13}C of studied corals (Host) and their Symbiodiniaceae (Symbionts) after a 5h pulse of $H^{13}CO_3^-$ and a 19h chase in filtered seawater. Data represent the mean ± standard error of five replicates collected from the shallow (8 m depth) and mesophotic (40 m depth) reef in Eilat, Israel. Nat. ab. = Natural abundance; reflects the natural abundance isotope composition prior to tracer incubation.

		Host			Symbionts		
		Nat. ab. (%)	Pulse (%)	Chase (%)	Nat. ab (%)	Pulse (%)	Chase (%)
Litophyton sp.	Shallow	1.0816 ± 0.0003	1.2272 ± 0.0205	1.2216 ± 0.0198	1.0844 ± 0.0002	1.2645 ± 0.0346	1.2986 ± 0.0325
	Mesophotic	1.0803 ± 0.0003	1.2449 ± 0.0175	1.2348 ± 0.0094	1.0823 ± 0.0005	1.3123 ± 0.0287	1.3021 ± 0.0279
Rhytisma fulvum fulvum	Shallow	1.0835 ± 0.0003	1.1730 ± 0.0065	1.1734 ± 0.0132	1.0904 ± 0.0008	1.1245 ± 0.0055	1.1026 ± 0.0029
	Mesophotic	1.0830 ± 0.0001	1.1617 ± 0.0142	1.1532 ± 0.0092	1.0957 ± 0.0018	1.1324 ± 0.0025	1.1242 ± 0.0066

Table S3: Total chlorophyll and autotrophic carbon acquisition of *Stylophora pistillata* along the depth gradient. A. Parameters normalized to surface area of the skeleton[4]. B. Parameters normalized to ash-free dry weight. The values of this table represent estimates calculated from A using a conversion coefficient (255.65 ± 8.98) determined in this study. P_C = autotrophic carbon acquisition.

**A**	Total chlorophyll ($\mu g\ cm^{-2}$)	P_C ($\mu g\ C\ cm^{-2}\ h^{-1}$)
Depth (m)	November	November
5	3.61	9.49
50	6.87	2.17

**B**	Total chlorophyll ($\mu g\ g^{-1}$ AFDW)	P_C ($\mu g\ C\ g^{-1}$ AFDW h^{-1})
Depth (m)	November	November
5	923	2,426
50	1,756	555

Table S4: Coefficients estimated to convert data normalized to surface area of the skeleton into data normalized to ash-free dry weight (mean ± standard error = 255.65 ± 8.98). The nubbins (n = 8) were fragmented from *Stylophora pistillata* colonies grown at the Monaco Scientific Center. DW = dry weight. AFDW = ash-free dry weight. Calculated coefficients are the ratio between surface area of the skeleton and ash-free dry weight.

Nubbin	DW (g)	AFDW (g)	Surface area (cm^2)	**Coefficient**
1	0.0609	0.0488	11.921	**244.416**
2	0.0610	0.0460	10.461	**227.373**
3	0.0992	0.0836	19.183	**229.524**
4	0.0855	0.0688	16.154	**234.830**
5	0.1341	0.0984	28.983	**294.543**
6	0.1071	0.0892	24.614	**276.018**
7	0.1112	0.0991	25.748	**259.860**
8	0.0158	0.0146	4.065	**278.638**

Supplementary figure

Figure S1: Levels of photosynthetically active radiation (PAR) received at the seasurface, 10 m depth and 40 m depth in Eilat during the five days of our experiment. Surface data was provided by the Israel National Monitoring program of the Gulf of Eilat and levels along the depth gradient were calculated according to Beer-Lambert law, with an attenuation coefficient of 0.1 m^{-1} [5,6].

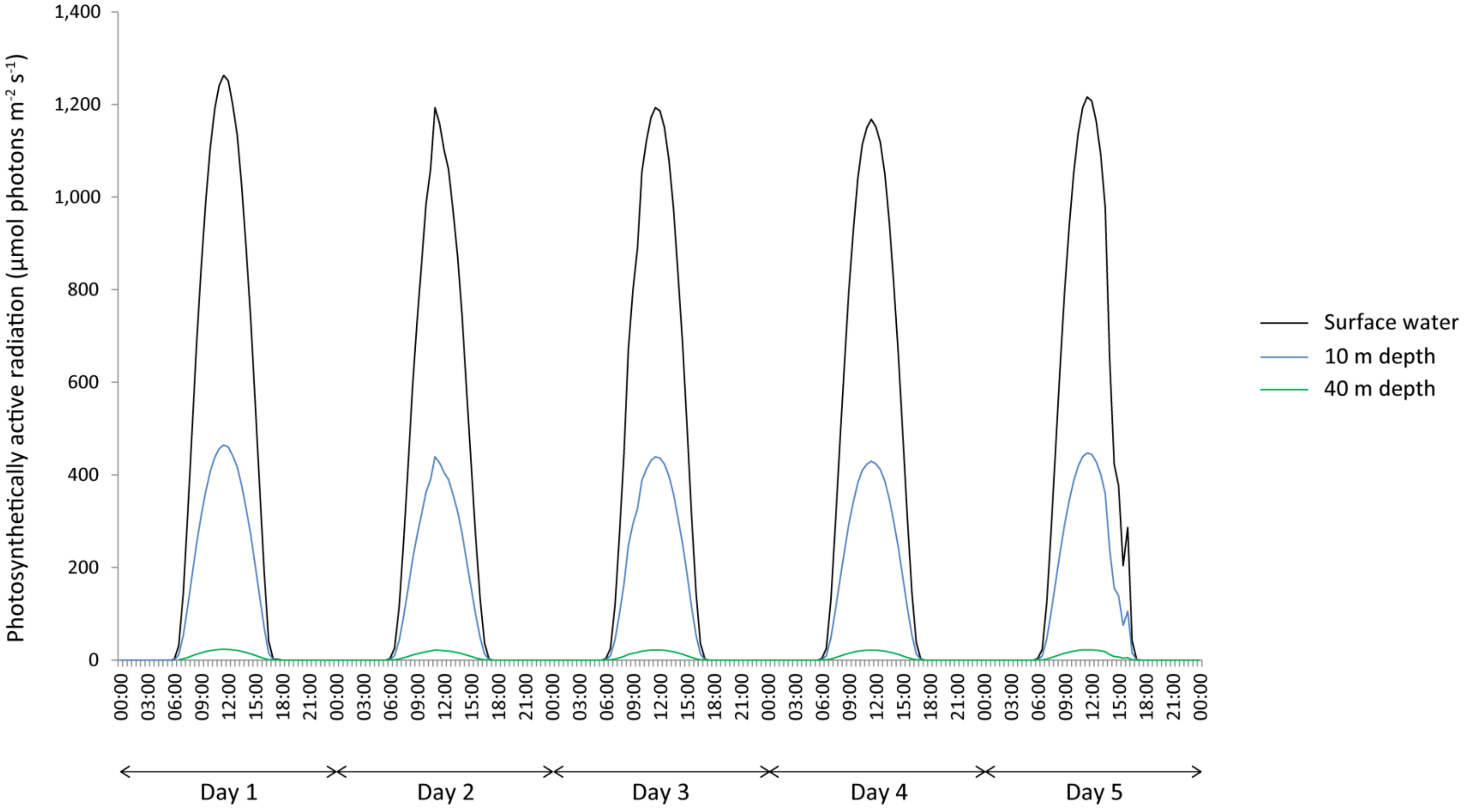

References cited in the Supplementary Information

1. Pupier, C. A., Bednarz, V. N. & Ferrier-Pagès, C. Studies with soft corals - recommendations on sample processing and normalization metrics. *Front. Mar. Sci.* **5,** 348 (2018).
2. Tremblay, P., Grover, R., Maguer, J.-F., Legendre, L. & Ferrier-Pagès, C. Autotrophic carbon budget in coral tissue: a new 13C-based model of photosynthate translocation. *J. Exp. Biol.* **215,** 1384–1393 (2012).
3. Jeffrey, S. W. & Humphrey, G. F. New spectrophotometric equations for determining chlorophylls a, b, c_1 and c_2 in higher plants, algae and natural phytoplankton. *Biochem. und Physiol. der Pflanz.* **167,** 191–194 (1975).
4. Ezzat, L., Fine, M., Maguer, J.-F., Grover, R. & Ferrier-Pagès, C. Carbon and nitrogen acquisition in shallow and deep holobionts of the scleractinian coral *S. pistillata*. *Front. Mar. Sci.* **4,** 102 (2017).
5. Akkaynak, D. *et al.* What is the space of attenuation coefficients in underwater computer vision? *Proc. - 30th IEEE Conf. Comput. Vis. Pattern Recognition, CVPR 2017* 568–577 (2017). doi:10.1109/CVPR.2017.68
6. Tamir, R., Eyal, G., Kramer, N., Laverick, J. H. & Loya, Y. Light environment drives the shallow to mesophotic coral community transition. *bioRxiv* 622191 (2019). doi:10.1101/622191
